# Massage Therapy for Neck and Shoulder Pain: A Systematic Review and Meta-Analysis

**DOI:** 10.1155/2013/613279

**Published:** 2013-02-28

**Authors:** Ling Jun Kong, Hong Sheng Zhan, Ying Wu Cheng, Wei An Yuan, Bo Chen, Min Fang

**Affiliations:** ^1^Yueyang Hospital of Integrated Traditional Chinese and Western Medicine, Shanghai University of Traditional Chinese Medicine, Shanghai 200437, China; ^2^Research Institute of Tuina, Shanghai Academy of Traditional Chinese Medicine, Shanghai 201203, China; ^3^Department of Orthopedics, Shuguang Hospital, Shanghai University of Traditional Chinese Medicine, Shanghai 201203, China

## Abstract

*Objective.* To evaluate the effectiveness of massage therapy (MT) for neck and shoulder pain. *Methods.* Seven English and Chinese databases were searched until December 2011 for randomized controlled trials (RCTs) of MT for neck and shoulder pain. The methodological quality of RCTs was assessed based on PEDro scale. The meta-analyses of MT for neck and shoulder pain were performed. *Results.* Twelve high-quality studies were included. In immediate effects, the meta-analyses showed significant effects of MT for neck pain (standardised mean difference, SMD, 1.79; 95% confidence intervals, CI, 1.01 to 2.57; *P* < 0.00001) and shoulder pain (SMD, 1.50; 95% CI, 0.55 to 2.45; *P* = 0.002) versus inactive therapies. And MT showed short-term effects for shoulder pain (SMD, 1.51; 95% CI, 0.53 to 2.49; *P* = 0.003). But MT did not show better effects for neck pain (SMD, 0.13; 95% CI, −0.38 to 0.63; *P* = 0.63) or shoulder pain (SMD, 0.88; 95% CI, −0.74 to 2.51; *P* = 0.29) than active therapies. In addition, functional status of the shoulder was not significantly affected by MT. *Conclusion.* MT may provide immediate effects for neck and shoulder pain. However, MT does not show better effects on pain than other active therapies. No evidence suggests that MT is effective in functional status.

## 1. Introduction

Massage therapy (MT), as one of the complementary and alternative treatments, is defined as a therapeutic manipulation using the hands or a mechanical device, which includes numerous specific and general techniques that are often used in sequence, such as effleurage (stroking), petrissage (kneading), and percussion [1]. It may be the earliest and most primitive tool to improve pain. The most ancient references to the use of massage come from China (around 2700 BC). With the popularity of MT in the world, common types of MT include Swedish massage, Shiatsu, Rolfing, reflexology, myofascial release, and craniosacral therapy.

With its popularity in pain relief, MT has become a widely accepted treatment for neck and shoulder pain. There are, however, inconsistent conclusions on effects of MT for neck and shoulder pain. Most prior reviews maintained that there was inconclusive evidence on effects of MT for neck and shoulder pain [2–4]. Others suggested that MT is effective for neck and shoulder pain [5, 6]. But in prior reviews, MT usually was viewed as an adjunctive therapy to prepare for mobilization, spinal manipulation, or other interventions. In addition, it was rarely employed as the main treatment method. Consequently, it is difficult to draw accurate conclusions regarding the effectiveness of MT when multiple treatments are involved. What is more, most of these reviews were outdated. 

Therefore, we performed an updated systematic review of all currently available data that included English and Chinese publications and conducted quantitative meta-analyses of MT on pain and functional status of patients with neck and shoulder pain to determine whether MT is a viable complementary and alternative treatment for neck and shoulder pain.

## 2. Methods

### 2.1. Search

We performed comprehensive computerized searches of the medical literature in 7 databases and reference lists through December 2011. English databases included PubMed, EMBASE, OVID-MEDLINE, and SPRINGLINK, and Chinese databases included China Knowledge Resource Integrated Database (CNKI), Weipu Database for Chinese Technical Periodicals (VIP), and Wan Fang Data. The main search terms were massage, manual therapy, Tuina, neck pain, neck disorders, cervical vertebrae, shoulder pain, and trapezius muscle. No restrictions on publication status were imposed. In addition, we performed hand searches at the library of Shanghai University of Traditional Chinese Medicine. 

### 2.2. Study Selection

Randomized controlled trials (RCTs) of MT for patients with neck and/or shoulder pain were included. There were no limitations on the participant's age, gender, or nationality. The focused intervention was MT using the hands or mechanical devices. The control intervention included placebo, a wait list control, no treatment, standard care, and any active treatments not related to MT. The main outcomes of interest were pain and functional status. We did not set any restriction on the type of tool used in the studies to measure these outcomes as there were no universally accepted tools available. We found various validated tools for these outcomes in different countries. The effects of MT included immediate effects (immediately after treatments: up to one day) and followup effects (short-term followup: between one day and three months, intermediate-term followup: between three months and one year, and long-term followup: one year and beyond).

Trials were excluded if any of the following were identified: (1) if neck and/or shoulder pain was caused by fractures, tumors, infections, rheumatoid arthritis, and so forth; (2) if MT was combined with other manual therapies including spinal manipulation, mobilization, chiropractic, and; (3) if controlled interventions also contained MT, since it would be impossible to evaluate the specific effect of MT; and (4) if the language was neither English nor Chinese. 

### 2.3. Data Abstraction

Two reviewers extracted data independently according to predefined criteria including the first author, the original country of the study, year of the study, pain location, pain duration, the sample size, the mean age of participants, the duration of treatments, the followup time, main outcome assessments, the intervention of experimental and control group, and main conclusion (mean improvement on pain). Any discrepancies were discussed until the authors reached consensus. 

### 2.4. Methodological Quality Assessment

The methodological quality of RCTs was assessed independently by two reviewers based on PEDro scale, which is based on the Delphi list and has been reported to have a fair-to-good reliability for RCTs of the physiotherapy in systematic reviews. This scale consists of 11 criteria: (1) study eligibility criteria specified, (2) random allocation of subjects, (3) concealed allocation, (4) measure of similarity between groups at baseline, (5) subject blinding, (6) therapist blinding, (7) assessor blinding, (8) less than 15% dropouts, (9) intention-to-treat analysis, (10) between-group statistical comparisons, and (11) point measures and variability data. Criteria (2)–(11) were used to calculate the PEDro score. Each criterion was scored as either 1 or 0 according to whether the criteria was met or not, respectively. The scores are summed, and a higher score represents a better methodological quality. A cut point of 6 on the PEDro scale was used to indicate high-quality studies as this has been reported to be sufficient to determine high quality versus low quality in previous studies [7]. If additional data or clarification was necessary, we contacted the study authors. And disagreements were resolved by discussions among the reviewers.

### 2.5. Data Synthesis and Analysis

The mean change in outcomes between the end of final intervention and the baseline was used to assess the difference between experimental group and control group in the meta-analyses. Standardised mean difference (SMD) was used because the studies included in meta-analyses assessed the outcome based on different scales (e.g., VAS 0–10 and VAS 0–100). And the SMD and 95% confidence intervals (CI) were calculated in the meta-analyses. For studies with insufficient information, the reviewers contacted the primary authors to acquire and verify the data when possible. In studies that involved more than one control group, the reviewers restricted our analyses to MT and each control group. In order to get more accurate heterogeneity we used random effects model employing variation factors among studies as correction weight. The *I* ^2^ was used to assess statistical heterogeneity. The reviewers determined that heterogeneity was high when the *I* ^2^ was above 75%. The Review Manager 5.0 was used for the meta-analyses. 

## 3. Results

### 3.1. Study Selection

**Figure 1:**
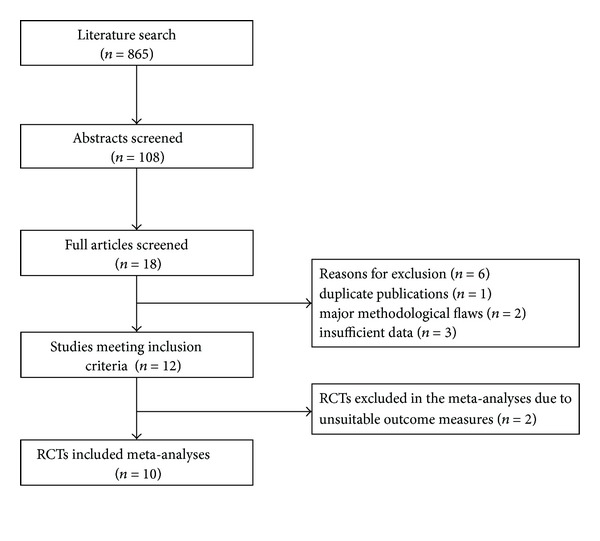
Study selection process. RCTs: randomized controlled trials.

We identified 865 abstracts from 7 English and Chinese databases. After initially screening 108 potentially relevant abstracts, we excluded 90 because they did not meet the inclusion criteria (e.g., systematic reviews, commentary, case report, technical report, not MT as a stand-alone treatment, participants were healthy, using psychosocial or biochemical outcome measures, and not in English or Chinese). We retrieved and reviewed 18 full articles. 12 RCTs [8–19] were eligible, including 10 English articles and 2 Chinese. In excluded studies, the trials were excluded due to duplicate publications (*n* = 1), major methodological flaws (*n* = 2), and insufficient data (*n* = 3). Two RCTs were excluded from meta-analyses due to unsuitable main outcomes [15, 17]. The study selection process is summarized in Figure 1.

### 3.2. Study Characteristics

Twelve eligible studies included 757 subjects with mean age of 42.3 ± 16.4, which, respectively, were conducted in US, Germany, Australia, UK, Spain, China, Thailand, and HongKong between 2001 and 2011. The disease duration ranged from 4 weeks to 10.4 years. The study duration lasted from 1 day to 10 weeks. The mean ± SD number of the session and treat time, respectively, were 7.0 ± 4.9 (range 1–18) and 26.6 ± 10.6 minutes (range 10–45 minutes). The followup time ranged from 3 days to 24 weeks.

Of twelve RCTs, 8 RCTs [8, 10, 13–17, 19] assessed the effectiveness of MT for neck pain and 4 trials [9, 11, 12, 18] for shoulder pain. MT in the studies included Chinese traditional massage, common Western massage, traditional Thai massage, slow-stroke back massage, soft tissue massage, manual pressure release, classical strain/counterstrain technique, and myofascial band therapy. The control therapies contained inactive therapies (waiting list control, standard care, and sham therapies) and active therapies including acupuncture, traction, physical therapy, exercise, and activator trigger point therapy. The characteristics of all studies are summarized in Table 1.

### 3.3. Methodological Quality

The quality scores are presented in Table 2. The quality scores ranged from 6 to 8 points out of a theoretical maximum of 10 points. The predetermined cutoff 6 was exceeded by all the studies included, indicating that all of them were considered to be of high quality; however, four articles were at the limit of the cutoff with scores of 6. The most common flaws were lack of blinded therapists (92% of studies) and blinded subjects (83% of studies). But this situation cannot be considered a flaw because blinded therapists are impossible, and blinded patients can be difficult in this kind of trials, and most studies used blinded assessors (92% of studies). Although all studies adopted random assignment of patients, six trials did not use adequate method of allocation concealment. Four studies were lacking of analysis by intention-to-treat analysis because they cancelled the dropout data in the last results. In other items on PEDro scale, the studies included showed higher methodological quality involving measure of similarity between groups at baseline, less than 15% dropouts, between-group statistical comparisons, and point measures and variability data. 

### 3.4. Quantitative Data Synthesis

#### 3.4.1. Immediate Effects of MT on Pain

Eight RCTs examined the immediate effect of MT for neck pain versus inactive therapies or active therapies. Six of them were included in the meta-analysis [8, 10, 13, 14, 16, 19]. The results showed that MT may have been more effective than inactive therapies, but there were no differences between MT and other active therapies. In two RCTs excluded in the meta-analysis, one [15] tested the effect of myofascial band therapy versus activator trigger point therapy or sham ultrasound. With regard to pain reduction, the results showed that the odds of patients improving with activator trigger point therapy was higher than patients treated with myofascial band therapy or sham ultrasound. The other RCT [17] assessed the effectiveness of MT for chronic neck pain compared with standard care. The author found that participants in the massage group experienced clinically significant improvements on neck disability index (39% versus 14% of standard care group) and on the symptom bothersomeness scale (55% versus 25% of standard care group).

Four trials tested the immediate effect of MT for shoulder pain versus inactive therapies [11, 12] or active therapies [9, 18]. All studies were included in the meta-analysis. The result showed that MT may have been more effective than inactive therapies, but there were no differences between MT and other active therapies.

**(1) MT versus Inactive Therapies**

**Figure 2:**
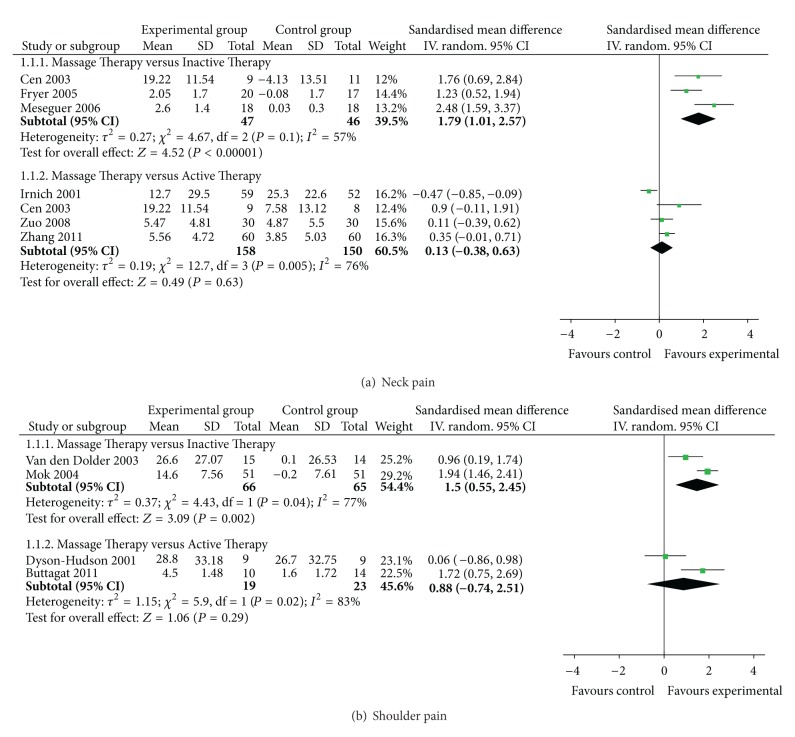
Forest plot of the immediate effect of massage therapy for (a) neck pain and (b) shoulder pain.

Three RCTs [10, 13, 14] assessed the immediate effect of MT on pain versus inactive therapies (including standard care or sham myofascial release) for neck pain. All studies showed significant effects of MT on pain relief compared with inactive therapies. The meta-analysis also showed favorable effects of MT (*n* = 93; SMD, 1.79; 95% CI, 1.01 to 2.57; *P* < 0.00001; heterogeneity: *χ* ^2^ = 4.67, *P* = 0.10, *I* ^2^ = 57%; Figure 2(a)). The study conducted by Irnich et al. [8] was excluded because it did not include the direct comparison between MT and sham laser acupuncture. Therefore, it is not possible to determine whether MT is superior to an inactive therapy.

Two RCTs [9, 12] compared the immediate effect of MT on pain with inactive therapies for shoulder pain. They reported favorable effects of MT on pain reduction. The meta-analysis also showed superior effects of MT on pain compared with inactive therapies (*n* = 131; SMD, 1.50; 95% CI, 0.55 to 2.45; *P* = 0.002; heterogeneity: *χ* ^2^ = 4.43, *P* = 0.04, *I* ^2^ = 77%; Figure 2(b)).

**(2) MT versus Active Therapies**

Four RCTs assessed the immediate effect of MT for neck pain compared with acupuncture [8], exercise [10], or traction [16, 19]. The meta-analysis did not show favorable effects of MT on pain reduction (*n* = 308; SMD, 0.13; 95% CI, −0.38 to 0.63; *P* = 0.63; heterogeneity: *χ* ^2^ = 12.70, *P* = 0.005, *I* ^2^ = 76%; Figure 2(a)).

Two RCTs tested the immediate effect of MT for shoulder pain compared with acupuncture [9] or physical therapy [18]. The MT group in two RCTs showed favorable effects versus control group. But the meta-analysis did not show superior effects of MT on pain reduction (*n* = 42; SMD, 0.88; 95% CI, −0.74 to 2.51; *P* = 0.29; heterogeneity: *χ* ^2^ = 5.90, *P* = 0.02, *I* ^2^ = 83%; Figure 2(b)).

#### 3.4.2. The Followup Effects of MT on Pain

**Figure 3:**
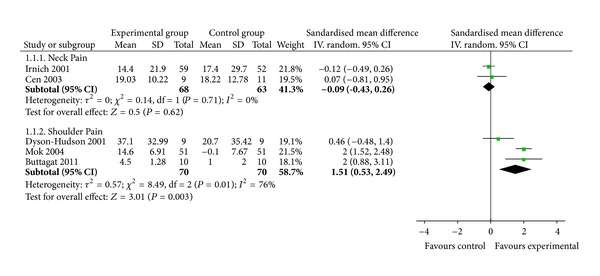
Forest plot of the followup effect of massage therapy for neck and shoulder pain.

In studies with followup, two RCTs assessed short-term effects of MT for neck pain. The authors reported that MT did not experience significant improvements on pain compared with acupuncture after a 12-week followup [8] or exercise after a 6-week followup [10]. The meta-analysis did not show significant effects of MT on pain in followup (*n* = 131; SMD, −0.09; 95% CI, −0.43 to 0.26; *P* = 0.62; heterogeneity: *χ* ^2^ = 0.14, *P* = 0.71, *I* ^2^ = 0%; Figure 3).

Three trials tested short-term effects of MT on shoulder pain compared with acupuncture [9], standard care [12], or physical therapy [18]. Two RCTs reported significant pain reduction, respectively, compared with standard care after 3-day followup [12] and physical therapy after a 2-week followup [18], while the other did not versus acupuncture after a 12-week followup [9]. The meta-analysis showed superior short-term effects of MT on pain reduction in followup (*n* = 140; SMD, 1.51; 95% CI, 0.53 to 2.49; *P* = 0.003; heterogeneity: *χ* ^2^ = 8.49, *P* = 0.01, *I* ^2^ = 76%; Figure 3).

#### 3.4.3. Effects of MT on Functional Status

**Figure 4:**
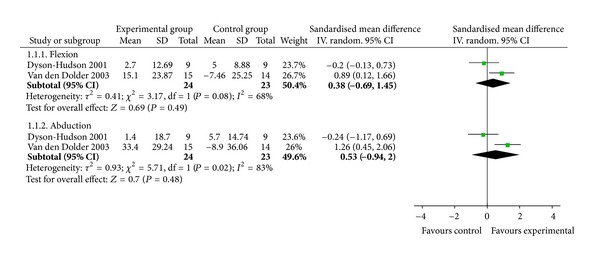
Forest plot of the effect of massage therapy in improving functional status related to shoulder pain.

Two studies tested the effectiveness of MT for shoulder range of motion compared with acupuncture [9] and waiting list control [11]. And the meta-analyses did not show significant immediate effects of MT on shoulder flexion (*n* = 47; SMD, 0.38; 95% CI, −0.69 to 1.45; *P* = 0.49; heterogeneity: *χ* ^2^ = 3.17, *P* = 0.08, *I* ^2^ = 68%; Figure 4) or shoulder abduction (*n* = 47; SMD, 0.53; 95% CI, −0.94 to 2.00; *P* = 0.48; heterogeneity: *χ* ^2^ = 5.71, *P* = 0.02, *I* ^2^ = 83%; Figure 4).

Six trials reported the effectiveness of MT on functional status of patients with neck pain; however, the quantitative meta-analysis had not been conducted due to the serious heterogeneity in assessment methods and ineligible reported data. Four of these studies reported favourable immediate effects compared with standard care [17], exercise (or standard care) [10], and traction [16, 19]. In the other two studies, MT did not show better immediate effects than acupuncture (or sham laser acupuncture) [8] or activator trigger point therapy [15]. Only two studies reported the followup effects of MT on functional status. One showed the short-term effects compared with exercise after a 6-week followup [10]. The other reported that MT showed less relapse rates compared with traction after a 24-week followup [19]. 

## 4. Discussion

The results suggested that MT may have been more beneficial than inactive therapies in immediate effects for neck and shoulder pain, but there were no differences between MT and other active therapies. On followup effects, the meta-analysis only showed the short-term benefit of MT for shoulder pain. With regard to the improvement of functional status, there was not valid evidence of MT for neck and shoulder pain.

We analyzed studies comparing MT with inactive therapies and active therapies separately because different control comparators address different questions. In addition, each control has advantages and limitations that must be considered in interpreting the analysis results. The inactive therapy control is intended to address the following question: is MT an effective therapy for neck and shoulder pain? Inactive therapies included standard care, waiting list, and sham treatment control in our review. Sham treatment control has advantages with regard to the blinding of patients, evaluators, or both to the treatment compared with other inactive therapies. The meta-analyses showed that MT is an effective therapy on relieving neck and shoulder pain. And the meta-analyses results from active therapy controlled RCTs address the question of whether MT is more effective than other active therapies for neck and should pain. No evidence suggested that MT was better than active therapies. In addition, we also paid attention to the immediate and followup effects of MT for neck and shoulder pain. 

Our positive results concur with those from systematic reviews. Ottawa panel evidence-based clinical practice guidelines [6] suggested that MT is effective for relieving immediate posttreatment neck pain symptoms, but data is insufficient to estimate followup effects. This systematic review included 5 RCTs published from 2003 to 2009, which demonstrated high methodological quality (≥3) according to the Jadad scale. And Tsao' systematic review [5] provided moderate support for the use of MT for shoulder pain, which qualitatively analyzed three RCTs of MT for shoulder pain. However, our systematic review included four new RCTs [14, 16, 18, 19] (three for neck pain and one for shoulder pain). Of notes, our review contained two Chinese RCTs [16, 19] of MT for neck pain with high methodological quality. And we quantitatively examined the effectiveness of MT for neck and shoulder pain. In our meta-analyses, we separately compared MT with inactive therapies and active therapies. We also paid attention to the immediate and followup effects of MT. So our update provides stronger evidence of MT for neck and shoulder pain. 

Our results differ from those of Ezzo and colleagues' systematic review [3] of MT for mechanical neck disorders, which concluded that the effectiveness of MT for neck pain remained uncertain. One suspected reason for this difference is that 6 high-quality RCTs [13–17, 19] have been published since 2003, of which 5 favored MT. Another possible explanation for the difference in the finding is that we used a different data analysis approach than Ezzo and colleagues. While we used meta-analyses, Ezzo' review declined to combine the trials because of trial heterogeneity. Any strictly qualitative approach may be problematic since it can be more subjective than meta-analyses. In addition, only six studies published from 1997 to 2003 examined MT alone as a treatment group in Ezzo' review. Four of those received low-quality scores. Two studies used treatments related to MT in control group. These were limited to evaluate the specific effect of MT because any individual study might affect the review's overall conclusions. More high-quality RCTs, classification of quantitative data synthesis, and the homogeneity of results of inactive therapy controlled RCTs and active therapy controlled RCTs in meta-analyses strengthen our confidence in our systematic review. 

### 4.1. Limitations of the Review

There are several limitations in our review. First, the distorting effects of publication and location bias on systematic reviews and meta-analyses are well documented [20, 21]. We are confident that our search strategy located all relevant studies. However, some degree of uncertainty remains. Another possible source of bias is that the more negative trials of MT for neck and shoulder pain may be never published in the peer-reviewed literature, so there were only two negative studies in our review [8, 15]. Our review also may be affected by the heterogeneity in characters of different MTs including frequency, duration, number of sessions, and massage technique. Our review contained many types of MT (e.g., Swedish massage, Chinese traditional massage, soft tissue massage, slow-stroke back massage, manual pressure release, myofascial band therapy, and traditional Thai massage). These are very different in characters of MT. As a result, the basic standard of MT is very important in further studies, especially in a mechanism of influence. In addition, there were less eligible trials due to strict eligibility criteria for considering studies in our review, so some meta-analyses were performed on the bases of two trials. It may influence combining results, but low eligibility criteria would generate more doubtful results. In order to get stronger evidence, we will update our review as soon as new eligible RCTs of MT for neck and shoulder pain are reported. 

### 4.2. The Possible Rationale of MT for Relieving Pain

Assuming that MT was beneficial on relieving pain related to the neck and shoulder, the complex interplay of both physical and mental modes may provide a possible rationale. MT delivered to soft and connective tissues may induce local biochemical changes that modulate local blood circulation, improve muscle flexibility, intensify the movement of lymph, and loosen adherent connective tissue, which may alternately improve reuptake of local nociceptive and inflammatory mediators [22]. These local effects may subsequently influence neural activity at the spinal cord segmental level, thereby modulating the activities of subcortical nuclei that influence pain perception [23]. 

## 5. Conclusion

MT is an effective intervention that may provide immediate effects for neck and shoulder pain. However, MT does not show better effects than other active therapies on pain relief. Additionally, MT only showed short-term effects for shoulder pain in followup. No evidence suggests that MT was effective in improving functional status related to neck and shoulder pain. 

Future studies of MT for neck and shoulder pain should adhere to large-scale and high-quality RCTs with long followup for better quantitative meta-analysis. Even though it is difficult to blind subjects and therapists for treatments, employing assessor blinding and allocation concealment are important for reducing bias. The RCT should adopt validated primary outcome measures, adequate statistical tests, applicable comparison groups, and standard MT. This comprehensive review of MT for neck and shoulder pain acts to provide guidelines for future researches.

## Figures and Tables

**Table 1:** Randomized controlled trials evaluating the effect of massage therapy for neck and shoulder pain.

First authors, year, country	Pain location, pain duration	Sample size, mean age (year),	Duration weeks	Follow-up weeks	Main outcome assessments	Experimental group intervention*	Control group intervention*	Main conclusion (mean improvements on pain)
Irnich 2001, Germany [8]	Neck pain 42% > 5 years	177 52	3	12	Pain VAS (0–100) Cervical mobility	Massage therapy (MT) (30 min/5 sessions)	(1) Acupuncture (AC) (2) Sham laser AC (30 min/5 sessions)	MT (12.70) < AC (25.30); MT (12.70) < Sham laser AC (19.20)

Dyson-Hudson 2001, US [9]	Shoulder pain 5.8 years	18 45	5	5	Pain WUSPI ROM	MT (45 min/10 sessions)	AC (20–30 min/10 sessions)	MT (28.80) > AC (26.70)

Cen 2003, US [10]	Neck pain NR	31 49	6	6	Pain NPQ (0–100) ROM	Chinese traditional massage (CTM) (30 min/18 sessions)	(1) Exercise (EX) (20 min/day) (2) Standard care (SC)	CTM (19.22) > EX (7.78) CTM (19.22) > SC (−4.13)

van den Dolder 2003, Australia [11]	Shoulder pain 28 weeks	29 64	2	—	Pain VAS (0–100) ROM	Soft tissue massage (STM) (15–20 min/6 sessions)	Waiting list (WL)	STM (26.60) > WL (0.10)

Mok 2004, Hong Kong [12]	Shoulder pain NR	102 73	1	3 days	Pain VAS (0–100) STAI	Slow-stroke back massage (SBM) (10 min/7 sessions)	SC	SBM (14.60) > SC (7.61)

Fryer 2005, Australia [13]	Neck pain NR	37 23	1 day	—	PPT	Manual pressure release (MPR) (1 session)	Sham myofascial release (SMR) (1session)	MPR (2.05) > SMR (−0.08)

Meseguer 2006, Spain [14]	Neck pain NR	54 40	1 day	—	Pain VAS (0–10)	Classical strain/counterstrain technique (CST) Modified strain/counterstrain technique (MST) (1 session)	SC	CST = MST (2.60) CST (2.60) > SC (0.03)

Blikstad 2008, UK [15]	Neck pain 4–12 weeks	45 24	1 day	—	Pain VAS (0–10) ROM	Myofascial band therapy (MBT) (1session)	(1) Activator trigger point therapy (ATPT) (2) Sham ultrasound (SU) (1 session)	MBT < ATPT MBT = SU

Zuo 2008, China [16]	Neck pain 10.4 years	60 42	2	—	Pain VAS (0–10) NDI	CTM (30 min/6 sessions)	Traction (TR) (20 min/14 sessions)	CTM (5.47) > TR (4.87)

Sherman 2009, US [17]	Neck pain 7.6 years	64 47	10	16	NDI CNFDS	MT (10 sessions)	SC	MT > SC (NDI)

Buttagat 2011, Thailand [18]	Shoulder pain 39 months	20 25	3	2	Pain VAS (0–10) STAI	Traditional Thai massage (TTM) (30 min/9 sessions)	Physical therapy (PT) (30 min/9 sessions)	TTM (4.50) > PT (1.60)

Zhang 2011, China [19]	Neck pain 1–3 years	120 23	10 days	24	Pain VAS (0–10) ASCS (0–29)	CTM (20 min/10 sessions)	TR (15 min/10 sessions)	CTM (5.56) > TR (3.85)

VAS: visual analog scale; WUSPI: wheelchair user's shoulder pain index; ROM: range of motion; NR: no reported; NPQ: Northwick park neck pain questionnaire; STAI: state-trait anxiety inventory; PPT: pressure pain threshold; NDI: neck disability index; CNFDS: Copenhagen neck functional disability scale; ASCS: Assessment scale for cervical spondylosis.

*Intervention/dose: number of intervention time/number of sessions.

**Table 2:** PEDro scale of quality for included trials.

Study	Eligibility criteria	Random allocation	Concealed allocation	Similar at baseline	Subjects blinded	Therapists blinded	Assessors blinded	<15% dropouts	Intention-to-treat analysis	Between-group comparisons	Point measures and variability data	Total
Irnich et al. [8]	1	1	0	1	0	0	1	1	1	1	1	7
Dyson-Hudson et al. [9]	1	1	1	1	0	0	1	1	0	1	1	7
Cen et al. [10]	1	1	0	1	0	0	1	1	0	1	1	6
van den Dolder and Roberts [11]	1	1	1	1	0	0	1	1	1	1	1	8
Mok and Woo [12]	1	1	0	1	0	0	1	1	0	1	1	6
Fryer and Hodgson [13]	1	1	0	0	1	0	1	0	1	1	1	6
Meseguer et al. [14]	1	1	0	1	0	1	1	1	1	1	1	8
Blikstad and Gemmell [15]	1	1	1	1	1	0	1	0	0	1	0	6
Zuo et al. [16]	1	1	0	1	0	0	1	1	1	1	1	7
Sherman et al. [17]	1	1	1	1	0	0	1	1	1	1	1	8
Buttagat et al. [18]	1	1	1	1	0	0	1	1	1	1	1	8
Zhang et al. [19]	1	1	1	1	0	0	0	1	1	1	1	7

0 = not meet the criteria; 1 = meet the criteria.
